# Barriers to knowledge sharing in Chinese healthcare referral services: an emergent theoretical model

**DOI:** 10.3402/gha.v9.29964

**Published:** 2016-02-15

**Authors:** Lihong Zhou, Miguel Baptista Nunes

**Affiliations:** 1 School of Information Management, Wuhan University, Wuhan, China; 2 School of Information Management, Sun Yat-sen University, Guangzhou, China

**Keywords:** Chinese healthcare systems, healthcare referral services, knowledge sharing, knowledge sharing barriers

## Abstract

**Background:**

This paper reports on a research study that aims to identify and explain barriers to knowledge sharing (KS) in the provision of healthcare referral services in Chinese healthcare organisations.

**Design:**

An inductive case study approach was employed, in which 24 healthcare professionals and workers from four healthcare organisations in the province of Hubei, Central China, were interviewed using semi-structured scripts.

**Results:**

Through data analysis, 14 KS barriers emerged in four main themes: interpersonal trust barriers, communication barriers, management and leadership barriers, and inter-institutional barriers. A cause–consequence analysis of the identified barriers revealed that three of them are at the core of the majority of problems, namely, the absence of national and local policies for inter-hospital KS, lack of a specific hospital KS requirement, and lack of mutual acquaintance.

**Conclusions:**

To resolve KS problems, it is of great importance that healthcare governance agencies, both at the national and regional levels, take leadership in the process of KS implementation by establishing specific and strong policies for inter-institutional KS in the referral process. This paper raises important issues that exceed academic interests and are important to healthcare professionals, hospital managers, and Information communication technology (ICT) managers in hospitals, as well as healthcare politicians and policy makers.

## Introduction

In modern healthcare environments, efficient healthcare referral services are indispensable for the provision of high quality, patient-centred healthcare services (1). Communication and knowledge sharing (KS) between healthcare professionals concerning individual patients that were referred from general practitioners (GPs) in community clinics in China to hospitals, or even between hospitals of different levels, are vital to ensure patients’ healthcare. This need to meet the demands, requirements, and needs of referral patients should be protected in the referral processes in any healthcare system (2, 3) and is not unique to the Chinese healthcare system.

KS has been widely discussed in healthcare environments. It has been universally agreed that appropriate KS processes, based on good practices of knowledge creation, storage, transfer, and utilisation, are fundamental to resolving daily medical problems challenging healthcare professionals and, more importantly, can dramatically improve the quality of healthcare services (4–6).

In healthcare referral services, it is of paramount importance that professionals communicate and share knowledge with each other to look after patients’ needs and healthcare requirements (2, 3). Without effective and efficient KS, healthcare referrals would merely be composed of bureaucratic procedures for handing over patients from hospital to hospital, and this procedural approach would contradict the principles of patient-centred healthcare (6, 7). This is exactly the current practice situation in Chinese healthcare referral services, which has been reported as very problematic, with the rich opportunity for KS between the various healthcare practitioners being largely neglected. For instance, Zhang et al. (8) investigated healthcare referral services in four Chinese cities: Wuhan, Enshi, Nanchang, and Shenzhen. According to their findings, 56% of hospital doctors never had any work-related interaction with GPs, whereas 57% of GPs never communicated with hospital doctors. Moreover, 61% of hospital doctors and 86% of GPs rated patient-centred KS as very poor (8). Ouyang (9) explained that hospitals and clinics are almost entirely isolated and have become individual information islands, on which the generation, storage, and utilisation of knowledge are completely independent and insulated.

This paper reports on a research project – supported by the National Natural Science Foundation of China – that aimed to investigate these severe problems of insufficient KS in referral practices. Specifically, the project aimed to identify, understand, and explain existing barriers to KS in the practice of referral services in the Chinese healthcare services. The researchers expect that this theoretical understanding and conceptual representation of barriers may serve as the basis of a re-evaluation of referral processes at the national level and the improvement of referral patient care.

## Methods

### Research aim and questions

According to the research aims stated above, three research questions were formulated to orient the research design and drive the process of theoretical model development:

RQ1: What are the barriers to KS in healthcare referral services in the Chinese healthcare system?

RQ2: What are the relationships between these barriers?

RQ3: How can the identified barriers form a coherent theory that can be used to improve the current situation in the Chinese healthcare referral system?

Because the Chinese healthcare system is characterised by very specific historical, cultural, social, political, and economic factors, it was decided not to adopt a deductive approach that might bias the study with a Western theoretical lens. Therefore, an inductive qualitative approach was adopted based on a thematic analysis methodology composed by an initial critical literature review of the Chinese context and a set of exploratory case studies. This process of literature review was undertaken to prompt theoretical sensitivity, as proposed by Strauss and Corbin (10), and enable the design of the semi-structured interview script used in the data collection process through the identification of major areas of interest to the study (11).

### Critical literature review

The research design was developed to apply a thematic analysis consisting of an intensive data collection process followed by analysis and theory formulation. The data collection consisted of interviews with healthcare professionals on both ends of the referral process. These interviews were conducted using semi-structured scripts. To design these semi-structured interview scripts, the researchers undertook a comprehensive critical literature review of Chinese healthcare referral services and healthcare KS in general.

Not surprisingly, an initial literature search indicated that healthcare referral and referral management in China have been extensively discussed in Chinese newspapers, academic journal articles, and dissertations, but seldom in English literature. This result led to the need to focus on Chinese literature and select the three major Chinese academic databases as sources: CNKI, Wanfang, and CQVIP. The literature search was performed in February 2014 using the following search terms and queries in Chinese (translated here into English for illustration purposes):

1. referra*
2. knowledge
3. information
4. management
5. communicat*
6. sharing
7. transfer
8. #2 OR #3
9. #4 OR #5 OR #6 OR #7
10. #8 AND #9
11. #1 AND #10
12. TIME = 2000–2014

The corresponding database search retrieved 948 articles overall, of which 693 articles were retrieved from CNKI, 95 from Wanfang, and 160 from CQVIP. After the titles and abstracts were reviewed and redundant articles manually excluded, 207 articles were finally included for the review. Papers were rejected for the following reasons:

- They did not specifically address the referral process.
- They were not from reputable academic sources.
- They focused only on technological issues (e.g. cloud, databases, electronic records) rather than on the referral process itself.
- They focused on discussions of US and European models without comparisons with the Chinese environment (there was a substantial number of such papers).

The critical literature review identified 11 KS barriers in three emerging themes, as shown in Table 1.

**Table 1:** Knowledge sharing barriers and themes that emerged from the literature review

Category	KS barriers
Communication issues	Patient records as ineffective KS tool
	Referral notes as ineffective KS tool
	Absence of referral information systems
Interpersonal issues	Inability to share knowledge to meet receiving professionals’ needs
	Inability to absorb knowledge received
	Lack of trust
	Lack of mutual acquaintance between healthcare professionals
Management and inter-organisational issues	Lack of explicit and pragmatic KS requirements
	Financial conflicts between healthcare organisations
	Neglect of tacit patient knowledge in current practices
	Overwhelmingly high workload

KS, knowledge sharing

These KS barriers and emerging themes were used as the basis for the design of the semi-structured interview script. Each of these early themes was operationalised into an interview question. The final script contained these 11 questions, two ice breaking questions, and a conclusion question. It was designed for interviews to last between 60 and 75 min.

### Selection of the multiple case studies

Case study approaches are very common and widely used research strategies in information and management sciences. A case study enables the investigation of contemporary phenomena in real-life contexts (12) and is useful for exploratory purposes and initiating a theory (13).

Because China is one of the largest countries in the world, with a population exceeding 1.3 billion, it would be virtually impossible to undertake a national study of the type proposed by this research. Moreover, the variety of contexts (social, economic, and even ethnic) would make it equally virtually impossible to generate a generic and generalisable theory that would encompass the whole nation. Consequently, and because this project aimed foremost at generating a first set of insights into this problem, a case study approach was selected.

The selection of case studies was based on the current structure of referral services in China, which has recently undergone significant changes. In fact, despite rapid economic growth in China, the current Chinese healthcare system fails to meet some of the population's basic needs (14). As reported by Yip and Hsiao (15), there are generally three primary discontents voiced by the public: the increasing and very pronounced inequality in healthcare accessibility between urban and rural areas; paid and, for many, unaffordable access to healthcare; and social impoverishment due to substantial medical expenses (commonly known in Chinese as *kan bing nan, kan bing gui*).

To resolve these problems, the Central Committee of the Communist Party of China and the State Council jointly announced a new wave of health reforms in April 2009, which ambitiously aim to achieve the universal provision of free or low-cost healthcare to the entire population by 2020 (16, 17). To ensure success, the Chinese government put forward a plan to increase annual spending from $357 billion in 2011 to $1 trillion in 2020 (16).

One of the key objectives of healthcare reform is to implement and operationalise a nationwide referral service to connect local healthcare organisations with mainstream hospitals (17). Ideally, this new referral service system is supposed to create efficient and seamless pathways to transfer patients to the most suitable healthcare facilities and specialists in a timely manner. Simultaneously it should become an effective KS channel to connect individual healthcare professionals in primary, secondary, and tertiary healthcare services (18). According to recent reports, the development of the referral system can be generally characterised as rapid and steady (19–21). In some major cities, such as Beijing, Shanghai, Guangzhou, Wuhan, Nanjing, and Shenzhen, the referral system has been successfully implemented (19, 21).

To define and understand the structure, connections, and relationships that characterise these primary, secondary, and tertiary healthcare services, a review of the grey literature was conducted, which consulted national and Hubei policy, as well as regulatory and governance documentation. The main source of information for national documentation was the Web repository for the National Health and Family Planning Commission of the People's Republic of China. Hubei-specific information was obtained from the Health and Family Planning Commission of the Hubei Province website. Finally, to understand the reality of practice of the referral system advocated in theory and policy, two hospitals and a community hospital community healthcare centre (CHC) were consulted. The researchers obtained access to regulatory and guidance documentation that fully defined how the referral system was put into practice and was operating at the moment.

**Fig. 1:**
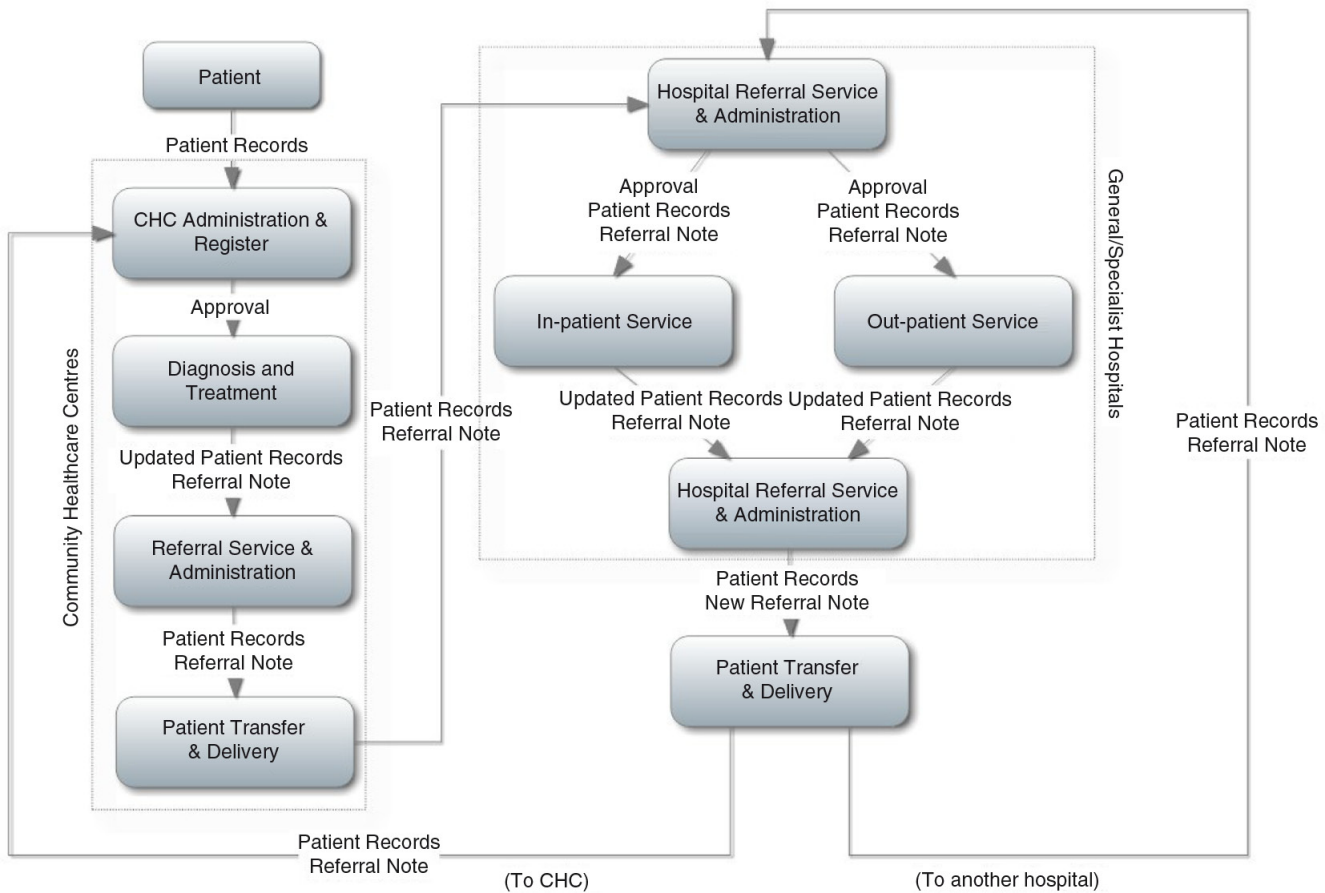
Healthcare referral procedural diagram.

This grey literature review enabled a good understanding of the operation of the healthcare referral system in the province of Hubei, as expressed in the healthcare referral procedural diagram presented in Fig. 1. This diagram illustrates a referral process that starts at the primary level. A patient is initially admitted and treated by GPs at CHCs. If the patient is diagnosed as requiring treatment in a general or specialist hospital, the CHC referral service and/or administration services of the community centre contact the receiving hospitals and arrange the necessary procedures and paperwork for patient transfer and delivery.

At the receiving hospital, a referral patient is initially received and admitted by the hospital referral administrative services. The patient is then assigned either to undergo further investigation of his or her condition or directly to specific treatment services, depending on the information received from the referring healthcare centre. After treatment, if the patient's major health problems have been resolved or effectively controlled, the patient is referred back to the CHC for recovery and rehabilitation treatment. On the other hand, if the patient's problems have not been resolved, the patient is referred to another hospital, perceived as more appropriate.

To reflect perceptions and views from all three levels of the Chinese healthcare referral system, four healthcare organisations located in Hubei Province, Central China, were selected as case studies:

- Tongji Hospital: a provincial hospital located in Wuhan, the capital city of Hubei. The hospital is highly reputable and arguably one of the best hospitals in China. Tongji Hospital is at the top end of referral services in Central China (Tier 3).
- Xiangyang Central Hospital: a regional central hospital located in the city of Xiangyang (approximately 280 km away from Wuhan), Hubei Province. This hospital provides the best healthcare services in the northwest region of Hubei and has emerged as the central link in the healthcare referral chain that connects top-level healthcare organisations, as well as community hospitals and clinics at lower levels in the city (Tier 3).
- Xiangyang Municipal Huimin Hospital: a community municipal hospital providing a full range of primary care services to nearly 12,000 people in the Tanxi area in the city of Xiangyang. The hospital is located approximately 3 km away from the Xiangyang Central Hospital (Tier 2).
- Wanshan Community Clinic: a small community clinic located in Wanshan Road in suburban Xiangyang. The clinic provides very basic and low-cost primary care services, including family, internal medicine, traditional medicine, rehabilitation, and emergency services to people living in adjacent areas. The clinic is operated and managed by the Xiangyang Municipal Huimin Hospital and has direct patient referral links with the hospital (Tier 1).

Moreover, there are two additional reasons for selecting these four specific healthcare organisations: 1) these institutions are located in neighbouring cities in Hubei and have established stable and close collaborative relationships, for which patients are frequently referred between them; and 2) the research teams obtained management support and guaranteed access to the informants in the organisations.

It is important to note here that the research design based on the four case studies presented in this paper is not aimed at performing a cross-case analysis as proposed by Yin (12) and Benbasat et al. (13). In this research, the four case hospitals were selected to allow for the views of the complete chain of healthcare referrals to be represented in the study.

### Data collection

Qualitative interview data were collected from the four case hospitals described above using semi-structured interview scripts and open-ended questions. These questions were derived from the literature review described above. Specifically, questions were designed from each of the KS barriers and themes that emerged from the literature review process (discussed above and listed in Table 1). Therefore, the study aimed to build on the existing body of in the field and, through theoretical sensitisation, avoid unnecessary processes of ‘reinventing the wheel’.

Twenty-four healthcare professionals, managers, and workers were approached and interviewed, as described in Table 2. The interviews were performed from March to May 2014 and lasted from approximately 40 to 80 min, individually. The principle of theoretical sampling, as normally used in grounded theory, was adopted in this study, because thematic analysis does not provide any guidance in this area. This theoretical sampling strategy closely connects the processes of data collection and analysis. The general aim here is to guarantee the richest possible variety of opinions and data on the phenomenon being studied. Therefore, there is an ongoing interplay between the collection and analysis of data, in which the collection of data is driven by the analysis, which starts as soon as the first bit of data is gathered (22). On the basis of the data analysis, the researcher articulates and derives indications for further data collection (23).

**Table 2:** Interview informants included in this study

Healthcare institutions included	Interview informants
Tongji Hospital	1 hospital manager, 2 doctors, 1 nurse, 1 ICT manager
Xiangyang Central Hospital	2 hospital managers, 5 doctors, 2 nurses, 1 ICT manager
Xiangyang Municipal Huimin Hospital	1 hospital manager, 2 general practitioners, 2 nurses, 1 ICT manager
Wanshan Community Clinic	1 manager, 1 general practitioner, 1 nurse

Securing access to potential interview participants has always been considered as one of the crucial issues in qualitative data collection (24). The researchers have experience of previous studies within the particular context of healthcare in China and were aware from the start of the strictly hierarchical, high power distance cultural trait (25) and the collectivist nature of the Chinese culture (11). Therefore, and knowing that subordinates and in-groups are usually dependent on power figures in Chinese organisations (26), the researchers were well aware that, if not asked by a senior health manager, healthcare professionals in China were not likely to volunteer readily or even agree to be interviewed. In the case of this research, the head of one of the hospitals selected as one of the case studies was approached directly and agreed to support the study. This very high-level individual then contacted the other hospitals and health centres using his ‘*guanxi*’ network. This is another fundamental Chinese cultural trait that represents a series of social interactions that form ‘intimate and reciprocal relations’ (27) in which favours and information are exchanged over time, enmeshing individuals within networks of reciprocal obligation (11). This phenomenon has become so important in China that ‘without *guanxi*, one simply cannot get anything done’ (28).

Potential interview participants were identified by this head of hospital and subsequently by other heads of the institutions selected. When more or different types of interviewees were required by the requirements of theoretical sampling, these directors were again approached for advice and further nomination of potential individuals.

Interviewees were asked to read and sign an interview consent form prior to the interview itself. During the early stage of each face-to-face meeting, the consent form was discussed and explained. Specifically, clear assurances concerning all aspects of confidentiality and anonymity were provided. There was a secondary aim for this face-to-face process of obtaining individual consent that consisted in seeking an opportunity for the research team to get to know interview participants personally and therefore establish an initial foundation of trust for the interview itself (11). This issue of trust became very important in terms of the data collection process itself in order to guarantee valid and reliable data (24).

**Table 3:** Interpersonal trust barriers and supporting interview quotations

Barriers	Supporting quotations
Lack of mutual acquaintance between healthcare professionals	‘We can talk freely if we know each other. I usually [feel more freely to] talk about what I think [about the patient], how I made my decision and arrived at my conclusion’. (13, p. 103) ‘If we know each other, I would know the doctor's reasoning logic, and what kind of information he [or she] would be expecting. Really, the communication is much shorter and easier, just right to the point’. (7, p. 45)
Lack of trust towards healthcare professionals at primary healthcare facilities	‘Treating patients and dealing with patients’ problems require personal experiences and a professional attitude. I would not say that doctors at small hospitals, a large number of them, are qualified’. (9, p. 37)
Lack of trust towards medical evidence produced in other healthcare facilities	‘We cannot accept the test results [medical evidence] transferred with referral patients. We usually ask the patient to retake all necessary tests. Because hospitals use different medical equipment, we don't know how accurate these tests are [in other hospitals]’. (13, p. 65)
Lack of trust towards tacit knowledge shared by peer professionals	‘Judgement and decision-making about a patient rely on a doctor's perception and subjective analysis. They are not always accurate. [In healthcare referrals], personal analysis can provide reference information. But we need to develop our own analysis’. (7, p. 50)
Belief in other parties’ tendencies to hide diagnosis and treatment errors	‘Sometimes, doctors and nurses in lower level hospitals may have made some mistakes or inappropriate delays when dealing with patient conditions and symptoms. When referring patients to us, they usually would not put the information into records or let us know. In these cases, we need to ‘reverse engineer’ what really happened back then’. (12, p. 43)

### Data analysis

Thematic analysis was used in this research as a systematic process of coding and representing data (29, 30) and aiming to seek and describe patterns across the qualitative data collected. In this research, interview data were examined, interpreted, coded, and constantly compared against themes and concepts. The starting point for this analysis, which was used to design the interview script, is expressed in Table 1. This initial framework enabled early identification of a set of KS barriers that were represented by codes, which were then used to further examine, label, and categorise valuable data segments that were identified in the subsequent interview data. Instead of just deductively verifying and validating the original set of KS barriers, these codes were used only at the data collection and early stages of the analysis process and then reconceptualised, expanded, and explained in detail according to the statements, interpretations, and perspectives of the healthcare practitioners interviewed. In addition, several new KS barriers emerged and were added to the emergent theory being established.

## Results

The findings for this research resulted in 14 KS barriers integrated into four main themes, which are all presented in detail in this section. Excerpts from the data are used to provide evidence and to enrich the theoretical narrative. All these excerpts are referenced as *number1*:*number2* in order to maintain anonymity. *Number 1* indicates the interview number and *number 2* refers to the paragraph of the interview transcript. Cultural aspects are referenced to illustrate the coding process.

### Confirmation of the process of KS

The data collected revealed that healthcare referral is a highly common procedure in Chinese hospitals. In the Xiangyang Municipal Huimin Hospital, ‘30% to 40% of patients will be referred to higher level hospitals’ (16, p. 12). Similarly, interviewed healthcare professionals at the Xiangyang Central Hospital stated that ‘two thirds of our patients were transferred from primary facilities’ (1, p. 16), whereas in Tongji Hospital, ‘the majority of the patients were from lower level hospitals’ (15, p. 16).

A healthcare referral is considered in any of the following three circumstances: first, ‘when it is judged that it is no longer possible to treat the patient [in the current facility], due to hardware problems, [that is] lack of appropriate diagnostic and treatment equipment’ (7, p. 11); second, ‘due to lack of necessary expertise or skills’ (7, p. 12); third, if ‘due to whatever reasons, a patient or [his or her] relatives explicitly requested a referral to another healthcare facility’ (12, p. 37). This last reason goes against the prescribed procedure described above, which states that a patient should not be referred if he or she can be treated in the current facility. However, this seems to be accepted as common practice in the case studies investigated. The reason behind this third referral option is the fact that patients are treated as paying customers; therefore, they may have a strong say in their choice of treatment.

According to the data collected, two professionals usually take the decisive role when deciding whether a patient needs to be referred to another facility, namely the doctor in charge and the head of the particular healthcare department. Both professionals need to agree and provide signatures on the patient records and a referral note. These are two documents that are mandatory in the transferral process and need to be with the patient and delivered to the receiving healthcare professionals.

**Table 4:** Communication barriers and supporting interview quotations

Barriers	Supporting quotations
Patient records as inadequate knowledge sharing tool	‘Patients are responsible to deliver their own medical records. Patient records have always been kept as classified documents, which are stored in the hospital archive. Before referral, patients can file formal application to photocopy their own records. It does not mean that you can photocopy everything [in the records]. The records are reviewed by the archive manager and can only be photocopied and prepared by one of the archive secretaries. Finally, the patient records need to be reviewed by the hospital management department and then marked with a hospital official stamp’. (1, p. 278)
Absence of communicating HIS between hospitals	‘The development of HIS in the hospital is solely sponsored and funded by the hospital management. [Therefore,] interconnections [between hospitals] clearly are not their priorities’. (8, p. 74)
Referral note as inadequate knowledge sharing tool	‘Usually doctors are not required to write a lot on a referral note, usually a sentence, no more than a paragraph’. (2, p. 108)
Absence of mechanism for informal KS	‘We usually communicate through telephone, before patient transfer. It is a personal communication channel, so that we do not record this. But the communication is rich, we can talk about anything about the patient. Sometimes we use email and Wechat [a Chinese smartphone instant messaging app] to send over CT and MRI images’. (1, p. 110)

At this stage, in some cases, the doctor in charge would contact the potential receiving doctors. However, this is not the standard procedure. Additionally, the communication is not for the purpose of KS, remaining at a superficial level merely ‘to make sure that the intended doctor agrees to take on the patient’ (13, p. 32). Once the referral is initiated, professionals on both ends are not required to communicate, either during or after the process of patient transfer.

The receiving healthcare professionals are ‘obliged to receive all patients, who are referred to [them]’ (5, p. 16) because ‘this is purely for the benefit of the patient being referred’ (15, p. 42). In defence of this practice, many interviewed healthcare professionals claimed that ‘communication is not always necessary because all the information we need is recorded in the patient records’ (1, p. 60). A few informants further noted that ‘only very occasionally do we need to talk to the previous doctors and to further clarify patient symptoms and problems’ (18, p. 49).

As emerged in the data analysis, instead of KS through personal and direct interactions, patient records and a referral note are the main vehicles and tools for KS. The two documents are expected to be transferred along with the patient throughout the entire referral process and until the patient fully recovers. Usually, the referral patient and relatives are responsible for preserving and delivering the two documents to the receiving professionals. However, as reflected in the data collected, the two documents cannot be considered as effective tools for KS and have in fact been identified as barriers, as discussed below.

Therefore, it has been confirmed that KS in the process of patient referral is, as anticipated in the literature review, extremely poor and solely based on very basic documents, containing very succinct technical information.

**Table 5:** Management and leadership barriers and supporting interview quotations

Barriers	Supporting quotations
Overwhelmingly high workload	‘We are just sometimes too busy to really communicate for every patient. Sometimes, [only] when I feel pressingly necessary, I will call the [referral] receiving doctor personally’. (7, p. 56)
Lack of specific hospital KS requirement	‘There is no management attention and specific regulations. No one is going to criticise you if you skip KS’. (20, p. 61)
Absence of in-hospital KS leadership	‘No department [in the hospital] is designated to lead and manage KS. In some hospitals, they have a Referral Management Office. In our hospital, referrals are managed and supervised by the General Management Office. I think they should take the leading role for KS’. (16, p. 93)

### KS barriers

Four main themes of KS barriers have emerged throughout the data analysis: interpersonal trust barriers, communication barriers, hospital management barriers, and inter-institutional barriers.

#### Interpersonal trust barriers

One of the aspects that often emerged from interviewees’ statements was the difficulty caused by a lack of trust between the intervenient parties in the referral process. This is a common problem in Chinese contexts, as effective communication can only occur if there is a relationship of trust between all parties engaged. As discussed frequently in the literature, information exchange is the primary motivation for communication and social relations (31–33). Chinese social networks have strict boundaries that define insiders and outsiders in relation to the individuals in the social network (33). Hwang (34) states that individuals are ‘not morally obligated’ to trust in those who are outsiders. Consequently, Scallon and Scallon (35) argue that individuals are less obligated to share important information with outsiders. However, and despite the strong contextualised nature of this study, there is evidence from the non-Chinese context that established that ‘in the absence of trust’ formal KS practices may be ‘insufficient to encourage individuals to share knowledge with others within the same work environment’ (36).

This aspect of the necessity of trust in KS also emerged very strongly from the data collected for this study and resulted in the main theme described in this section, which encompasses five barriers:

- Lack of mutual acquaintance between healthcare professionals
- Lack of trust towards healthcare professionals at primary healthcare facilities
- Lack of trust towards medical evidence produced in other healthcare facilities
- Lack of trust towards tacit knowledge shared by peer professionals
- Belief in the other party's tendency to hide diagnosis and treatment errors

From the data analysis, it was clear that interpersonal trust is a key success factor to activate spontaneous KS in the process of patient referral in the context of Chinese healthcare services. As revealed by analysis of the collected data, mutually acquainted healthcare professionals are more likely to engage in active KS and more freely and openly share personal understanding, perceptions, and opinions about a referral patient.

However, a mutual acquaintance does not always exist in every patient referral. In truth, as asserted by a number of informants, in most cases, the healthcare professionals at the two sides of a patient referral do not have a previously established acquaintance and therefore do not trust each other in the sense discussed above. In the reality of practice, KS is most likely to be ‘neglected’ (18, p. 48) because ‘[without an acquaintance], we do not even have a telephone number to begin with’ (1, p. 157), and ‘the conversation is unlikely to be taken seriously and is usually kept very brief’ (13, p. 103). It is particularly problematic when ‘a healthcare referral requires interdisciplinary specialists at both ends’ (18, p. 64).

However, this lack of trust may have roots in equally prevalent professional prejudices. As reflected in the data collected, healthcare professionals who worked at higher-level, larger-scale healthcare organisations expressed clear distrust of fellow doctors, GPs, and nurses at lower-level hospitals and community healthcare services at the primary level. Many hospital specialists we interviewed explicitly expressed their prejudices by stating that community healthcare professionals are ‘just general practitioners’ (2, p. 232) and ‘do not have high [competent] medical skills’ (3, p. 231). These statements clearly indicate untrusting relationships between healthcare professionals, which result in mistrust of the knowledge being shared by professionals working at lower-level healthcare organisations.

**Table 6:** Inter-institutional barriers and supporting interview quotations

Barriers	Supporting quotations
Absence of political requirement for inter-hospital KS	‘The government probably wants to put forward KS between healthcare professionals and between hospitals. But we receive no specific guidelines on what should we do exactly’. (1, p. 28)
Financial conflict between hospital management	‘Patients represent profits. I am sure the majority of healthcare professionals have their heart in the right place. But there are some cases in which hospitals just do not let patients go. I encountered several cases where they insisted on performing surgical procedures to remove brain tumours, even though they did not have adequate skills and equipment to do so. Then, things got out of hand and they finally decided to transfer the patient to us’. (1, p. 17)

Another prevalent professional prejudice is the lack of trust in medical evidence and test results produced and shared by other hospitals. The lack of trust is particularly severe when medical evidence is produced in community facilities and services, where, as many interviewed hospital specialists asserted, ‘the equipment is usually out-dated’ (6, p. 173), ‘poorly maintained’ (11, p. 82), and ‘they do not have the capacity to take care of a patient with severe conditions’ (6, p. 175). One of the interviewed hospital doctors directly expressed his distrust by stating that ‘the test results they provided down there … well … there are probably no nice words to qualify it … we don't take them very seriously up here’ (1, p. 184). In this case, even the sharing of explicit knowledge can be observed as unnecessary in the healthcare referral process.

Furthermore, tacit knowledge is taken even less seriously when compared with explicit knowledge. Tacit knowledge in this context usually consists of the healthcare professionals’ personal experience, perceptions, and judgements, which accumulate through processes of dealing and interacting with individual patients, their families, and their communities. Therefore, the sharing of tacit knowledge should be observed as equally important. Nonetheless, as shown in the data gathered, because the experience, competence, and professional decisions of others are not trusted, tacit knowledge is observed and generally considered as not reliable and is often ‘discarded’ (4, p. 104) by the receiving professionals.

Another problem for KS that emerged from the analysis was the perception that, in some cases, Chinese healthcare professionals may tend to hide any information that may have led to previous errors and exclude it from the official records. Understandably, previous problems and mistakes can be extremely important for the remainder of the patient's treatment. If not verified, these problems are passed to the receiving institutions, which are then held responsible.

Sometimes, doctors and nurses in lower level hospitals may have made some mistakes, or inappropriate delays, when dealing with patient conditions and symptoms. When referring patients to us, they usually would not put the information into records or let us know. In these cases, we need to ‘reverse engineer’ what really happened back then. (12, p. 43)

Similarly, the referring party may be very reluctant to send detailed information because they may be blamed themselves, as expressed by one of the interviewed healthcare professionals, who claimed that this type of information is ‘too sensitive [to share]’ (20, p. 124) to ‘avoid being criticised and held accountable, if anything goes wrong [after referral]’ (9, p. 37).

Supporting interview quotations for the discussion of the interpersonal trust barriers are shown in Table 3.

#### Communication barriers

As revealed by the data gathered from the four case studies, the KS problems are further compounded by inadequate channels for communication. Specifically, the data analysis points to four barriers to KS:

- Patient records as inadequate KS tools
- Referral notes as inadequate KS tools
- Lack of communicating Hospital information systems (HIS) between hospitals
- Absence of a mechanism for informal KS

As observed during data collection in the field, nearly all interviewed doctors had a computer terminal at their desk. Two hospitals, namely the Tongji Hospital and the Xiangyang Central Hospital, were in the process of implementing hospital information systems and had fully implemented electronic patient records systems. However, the existing systems had a limited capacity for inter-hospital communication and KS. The two fully operationalised patient records systems were not interconnected and were thus unable to transfer and share any information in digital form. In fact, when referring a patient, all records needed to be printed on paper and then hand-delivered by the referral patient to the receiving institution.

Paper-based patient records are not really effective or adequate for KS. Many interviewed professionals stated that patients and their relatives usually cannot properly store and preserve the patient records, and ‘only about 20% to 30% patients can bring along their [complete] patient records’ (2, p. 34). Moreover, hospitals are very aware that patient records can reveal previous errors and mistakes. Therefore, patient records are usually ‘thoroughly and carefully reviewed for nearly a month’ (14, p. 28) ‘by the hospital management department’ (3, p. 91), before being handed over to the patient being referred. In this case, not only is the sharing of knowledge and information critically delayed, but the value of the patient record is also reduced due to redactions and censorship.

A referral note is another paper-based document, which emerged negatively in the analysis and was not originally designed for the purpose of KS. In reality, a referral note is ‘only a standardised and structured paper form, which records very generic information about the patient, such as, name, gender, age and the reason for referral’ (2, p. 188). Furthermore, the referral note is not really used for communication and KS but as an administrative document and as evidence for ‘when a patient needs to be reimbursed for healthcare expenses from the healthcare insurance account in different hospitals’ (16, p. 31).

There is strong evidence in the knowledge management (KM) literature that knowledge shared through formal channels mainly tends to be explicit in nature (36–38). Patient records and a referral note are formal documents, contain explicit knowledge, and are particularly suited for formal channels. However, they proved to be particularly inefficient in supporting KS in this case. The data analysis also provided indications of the potential of informal channels for KS in the context of this study. This finding is also supported by KS research. For instance, Stevenson and Gilly (39) proposed that ‘even when clearly designated channels of communication exist in organisations, individuals tend to rely more on informal relationships for communication’. Evidence in support of this statement was also found in the present study.

The data analysis showed that telephone communication is perceived to be a more convenient and flexible channel and is widely used. Email and instant messaging are also very commonly used channels for sharing patient knowledge in practice. Nonetheless, evidence shows that informal communication is based on a mutual relationship and interpersonal trust that were previously established. Moreover, due to an absence of intra- and inter-hospital KS mechanisms and without an explicitly defined code of practice, informal communication usually occurs when a doctor ‘feels it is necessary’ (5, p. 52) and relies on ‘personal perception on what should be talked about over the phone’ (1, p. 152). Therefore, without previously acquired trust relationships, it seems very unlikely that any informal channels of KS may ever be used.

Supporting interview quotations for the discussion of the communication barriers are shown in Table 4.

#### Management and leadership barriers

The data analysis further underscored that KS has become particularly problematic due to a lack of clear KS hospital management policies and leadership. In fact, previous research has identified that within Chinese hospital environments, there is a need to formalise the processes of intraprofessional collaboration and formally regulate activities of intraprofessional communication and the sharing of patient knowledge (6, 22). The lack of such strict formalisation severely hinders KS, as demonstrated in this study, which is mainly due to the following:

- Overwhelmingly high workload
- Lack of specific hospital KS requirements
- Absence of in-hospital KS leadership

When collecting data in the field, the research team had opportunities to see inside the case study hospitals. It was easy to observe that all the hospitals investigated were overly crowded with patients. The healthcare professionals were extremely busy and had very high workloads; therefore, they were usually ‘more concerned with solving the patient's immediate problems’ (13, p. 51) and were prone to ‘under-prioritise necessary communication and KS’ (17, p. 176). In truth, KS is perceived by these overworked practitioners as an additional layer of administrative complication in their already overwhelmingly busy daily routines.

Moreover, KS might not always have been considered as important or even necessary by the healthcare professionals interviewed because there was ‘no explicit hospital requirement’ (4, p. 128) to do it. As a consequence, KS ‘seems less important in practice’ (12, p. 48), ‘not mandatory’ (13, p. 82), and therefore ‘not important’ (13, p. 82). In fact, in the case hospitals investigated, despite a very general management statement that declared the need for KS, there were no well-established, defined, and implemented KS policies or requirements. More importantly, there were no designated KS managers and no supporting staff for this type of sharing activity. Consequently, communication was solely based on an ‘individual professional's personal conscience and sense of responsibility towards the patient’ (12, p. 60).

Supporting interview quotations for the discussion of the management and leadership barriers are shown in Table 5.

#### Inter-institutional barriers

The last main theme to emerge from the data analysis refers to KS barriers that result from negative influences brought by difficult and complex inter-hospital relationships:

- Absence of national and local policies for inter-hospital KS
- Financial conflicts between healthcare organisations

Interviewees indicated that the lack of KS within the hospital environment results from the absence of ‘clear guidelines [established] by the government’ (5, p. 105), and thus no real efforts have been made in articulating practical and specific KS requirements and regulations between the different healthcare institutions by their respective hospital management.

Furthermore, the data collected revealed inter-institutional financial conflicts that have resulted in communication and KS problems. As explained by the interview informants confirming the literature review findings, the Chinese central government decided to push the healthcare industry into a free market system in the early 1980s. This change in policy meant that the central government significantly reduced financial support to healthcare organisations. Instead, healthcare organisations and practitioners were expected and forced to generate their own financial revenue, mainly through patient charges based on the provision of health services. A few interview informants revealed that some hospitals (not disclosed here for ethical reasons) have established tight control on referring out patients to retain and increase financial gains, even when a referral would be of evident benefit to a patient. On an even worse note, in some cases, if a patient insists on being referred to another healthcare facility, doctors can ‘refuse to provide patient records and any supporting documentation’ (16, p. 90). Clearly, post-1980s financial struggles and the need for self-financing have created inter-institutional tension, conflict, and competitive relationships between hospitals. In some cases, these seem to have degenerated into unethical violation of patient-centred principles, which also hinders and prevents active KS in healthcare referrals.

Supporting interview quotations for the discussion of the inter-institutional barriers are shown in Table 6.

## Discussion

**Fig. 2:**
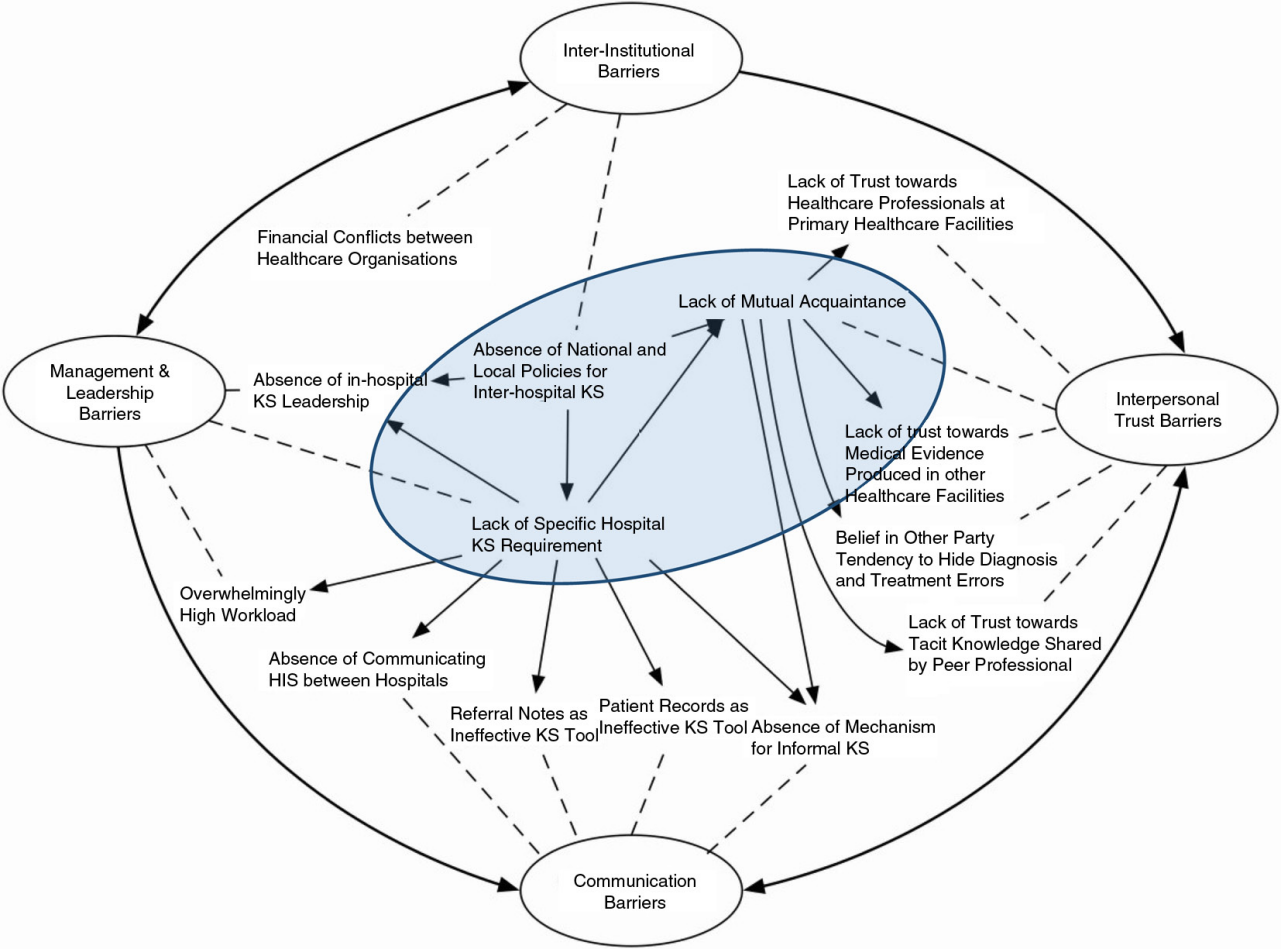
A model of knowledge sharing barriers, relationships, and themes.

The main analytical tool for this research was thematic analysis. This type of inductive approach is very useful for producing a list of themes, which can then be very easily expressed in terms of a structured theoretical narrative such as the one presented in the previous section. However, this study aimed at reaching further to propose a model of barriers that may be used in the future to resolve the problems encountered. Therefore, the data were re-analysed to understand the relationships between the themes identified from the interviewees’ perspectives. This process resulted in the conceptual model presented in Fig. 2.

As shown in Fig. 2, there are three types of relationships. First, the solid single-arrow lines represent the cause–consequence relationships between individual KS barriers. Second, the dotted lines demonstrate the relationships between the barriers and the emerging themes. Finally, the bold arrow lines exhibit the relationships between the four emerging themes, which are shown in ovals.

The four theoretical themes discussed above (shown in Fig. 2 as the outer layer) are interconnected, which is mostly caused by management and inter-organisational barriers. Moreover, the KS barriers identified are interconnected, and some are mutually influential. It is also important to note that the four outer themes can be observed as transferable to any context other than the Chinese one. The barriers identified at this level are generic and potentially recognisable in any other healthcare environment. However, upon close inspection, it is apparent that the causes for these barriers are very specific to the Chinese context. The three core causes for the barriers are all specific to the Chinese healthcare environment and represent unique Chinese cultural and governance traits discussed in the previous section:

- The national problem related to the lack of clear inter-institutional KS
- The organisational problem created by the absence of clear guidelines and regulations for KS in the hospital
- The individual problem caused by Chinese cultural traits associated with the need for trust before meaningful KS

This triangle of national, organisational, and individual barriers is at the centre of the specifically Chinese problem in the referral process and is deliberately represented by the main barriers highlighted in blue in the centre of Fig. 2. Resolving these problems is certainly not an easy proposition, but if changing Chinese cultural traits related to trust is virtually impossible, changing governance at both the national and hospital levels should be feasible. Therefore, it is of paramount importance that government healthcare agencies, at both the national and regional levels, take the lead in changing this process by establishing clear and strong policies for inter-institutional KS in the referral process. The creation and enforcement of such policies will in turn force hospitals to conform and implement their own regulations for KS. These regulations will then force individuals to overcome their cultural reluctance and engage in effective and productive KS.

## Conclusions

This research study aimed to identify barriers to KS in Chinese healthcare referral services. The study selected four healthcare institutions located in Hubei Province, Central China, as research case studies. It became clear in this study that despite clear (but not well-implemented, monitored, or controlled) political requirements for inter-institutional KS, the referral process in the Chinese healthcare system still suffers from severe problems. This issue does not derive from a lack of awareness of the importance and value of KS by practitioners but rather from a combination of a lack of governance and adverse cultural traits. As proposed above, the resolution of this problem lies in national and regional leadership, which needs to establish clear governance of the KS process and force the organisational and individual layers to conform.

The authors are aware that this is a misleadingly simple solution. Establishing specific and pragmatic strategies to resolve the KS barriers identified and to improve KS in Chinese healthcare referral services will require consultation, negotiation, and strong leadership, as well as political will.
